# The proteins of iodine metabolism in the pathophysiology of the thyroid gland

**DOI:** 10.1186/1756-6614-6-S2-A13

**Published:** 2013-04-05

**Authors:** Barbara Czarnocka

**Affiliations:** 1 Department of Biochemistry and Molecular Biology, The Centre of Postgraduate Medical Education, Warsaw, Poland

## 

Iodide (I-) is a trace element (0.0001% lithosphere) that is an important constituent of thyroid hormones. The iodide-containing T3 and T4 are crucial for normal development and the proper functioning of numerous metabolic pathways in probably all adult tissues. The biosynthesis of T3 and T4 involves thyroid specific proteins, found predominantly but not exclusively in the thyroid tissue. The process requires the presence of iodide (I-), a peroxidase (TPO), a supply of H2O2, and an iodine acceptor protein (Tg). The active trapping I- from the blood by basolateral iodide transporter NIS and concentration in the thyroid gland is a first step of hormonogenesis. Then I- is translocated across apical membrane to the follicle lumen by apical anion transporter pendrin. Once the I- reaches the colloidal lumen it is quickly oxidized by TPO, the key enzyme of biosynthesis. Thyroperoxidase is localized as a dimer at the apical membrane – colloid interface where the catalytic sites are exposed to the colloidal lumen, and where the main steps of hormonogenesis take place. Then oxidized iodide is further bound to tyrosyl residues in Tg – a precursor and storage form of thyroid hormones. The hormonogenic monoiodotyrosine (MIT) and diiodotyrosines (DIT) are subsequently coupled to form of T3 and T4. These key proteins engaged in the process of thyroid hormone synthesis are involved in pathological autoimmune response and genetic abnormalities in each of these proteins are responsible for some of thyroid disorders.

